# *C. elegans* feed yolk to their young in a form of primitive lactation

**DOI:** 10.1038/s41467-021-25821-y

**Published:** 2021-10-05

**Authors:** Carina C. Kern, StJohn Townsend, Antoine Salzmann, Nigel B. Rendell, Graham W. Taylor, Ruxandra M. Comisel, Lazaros C. Foukas, Jürg Bähler, David Gems

**Affiliations:** 1 Institute of Healthy Ageing, and Research Department of Genetics, Evolution and Environment, University College London, London, WC1E 6BT UK; 2 Molecular Biology of Metabolism Laboratory, The Francis Crick Institute, London, NW1 1AT UK; 3 Wolfson Drug Discovery Unit, Centre for Amyloidosis and Acute Phase Proteins, Division of Medicine, University College London, London, NW3 2PF UK

**Keywords:** Evolutionary theory, Genetics

## Abstract

The nematode *Caenorhabditis elegans* exhibits rapid senescence that is promoted by the insulin/IGF-1 signalling (IIS) pathway via regulated processes that are poorly understood. IIS also promotes production of yolk for egg provisioning, which in post-reproductive animals continues in an apparently futile fashion, supported by destructive repurposing of intestinal biomass that contributes to senescence. Here we show that post-reproductive mothers vent yolk which can be consumed by larvae and promotes their growth. This implies that later yolk production is not futile; instead vented yolk functions similarly to milk. Moreover, yolk venting is promoted by IIS. These findings suggest that a self-destructive, lactation-like process effects resource transfer from postreproductive *C. elegans* mothers to offspring, in a fashion reminiscent of semelparous organisms that reproduce in a single, suicidal burst. That this process is promoted by IIS provides insights into how and why IIS shortens lifespan in *C. elegans*.

## Abstract (web summary)

It is unclear why *C. elegans* continues to produce large quantities of yolk after reproduction. Here the authors show that post-reproductive *C. elegans* mothers vent yolk which supports their offspring’s growth, serving as a form of primitive lactation.

## Introduction

Adult *C. elegans* hermaphrodites exhibit severe senescent pathology that begins to develop within days of reaching sexual maturity^1–4^. For example, after depletion of self-sperm, intestinal biomass is converted in an autophagy-dependent manner into yolk, leading to intestinal atrophy and yolk steatosis (pseudocoelomic lipoprotein pools, PLPs)^1–3,5,6^. These senescent pathologies are promoted by insulin/IGF-1 signalling (IIS), which also shortens lifespan^3,7^. This pattern of rapid and severe pathology in organs linked to reproduction is reminiscent of semelparous organisms where massive reproductive effort leads to rapid death (reproductive death) as in Pacific salmon^8,9^. Moreover, self-destructive conversion of somatic biomass to support reproduction, often involving autophagic processes, is a characteristic feature of reproductive death^8^. Yet arguing against the occurrence of reproductive death in *C. elegans* is the apparent futility of post-reproductive yolk production. Here we show that this effort is not futile, since post-reproductive mothers vent yolk through their vulva, which is consumed by progeny and supports their growth and fertility; thus vented yolk serves a function similar to milk, and *C. elegans* mothers exhibit a form of primitive lactation that is coupled to senescence. Moreover, wild-type IIS promotes lactation, where maternal soma is consumed to support resource transfer from sperm-depleted mothers to larval kin, thereby accelerating aging.

## Results

### Post-reproductive *C. elegans* hermaphrodites vent yolk

**Fig. 1:**
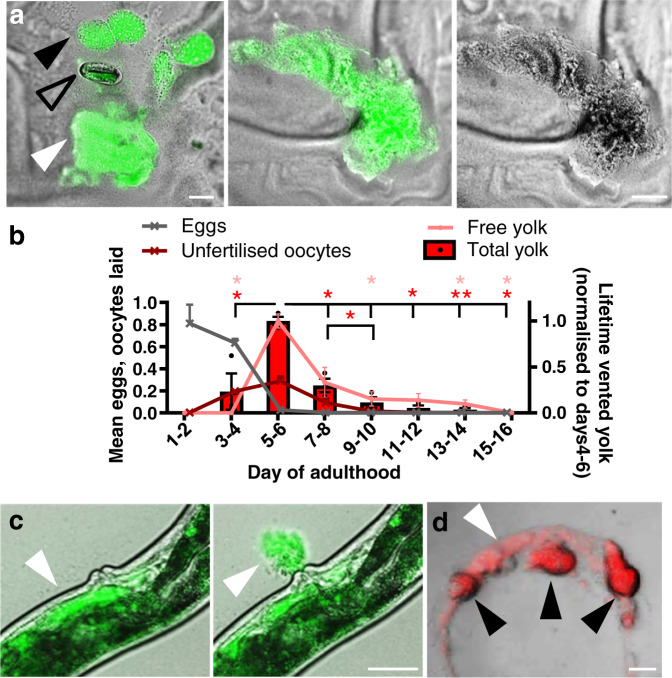
Post-reproductive *C. elegans* hermaphrodites vent yolk. **a** Vented yolk pools and unfertilised oocytes on culture plates from hermaphrodites on d4 of adulthood, expressing *vit-2::GFP*. For other examples and separate images for Nomarski and epifluorescence microscopy see Supplementary Fig. 1c. Scale 50 μm. **b** Lifetime reproductive schedule, oocyte production, and proportion of total vented yolk (from oocytes + free yolk) and free yolk quantitated from VIT-2::GFP on plates and normalised to days 4–6. Trails were started at the L4 stage, and day 1 as specified denotes the first 24 h after the L4 stage, which includes part of day 1 of adulthood. Mean ± S.E.M. of 3 trials displayed (*n* = 50 worms per trial for venting and 10 per trial for brood sizes). *P < 0.05, **P < 0.01 by one-way ANOVA (Tukey correction; statistical tests performed on raw data), compared to day 4–6; red, total yolk (free + oocytes); pink, free yolk alone. Total yolk left to right compared to d5-6 for stars *P* = 0.010, 0.027, 0.024, 0.004, 0.011; d7–8 vs 9–10 *P* = 0.047. Free yolk left to right *P* = 0.016, 0.019, 0.017, 0.017. **c** Yolk vented through the vulva of a day 4 adult. Live imaging of VIT-2::GFP day 4 adults performed with yolk initially present in the uterus (left) and then seen vented 7 sec later (right) (white arrowhead). For presence of yolk in the uterus, other examples of yolk venting, time series, and comparison to egg laying on day 2 of adulthood see Supplementary Fig. 1d–f and Supplementary Movie 1. Scale 50 μm. **d** Lipid in vented yolk and unfertilised oocytes from day 4 adults subjected to vital staining with the lipid dye Bodipy 493/503. Scale 50 μm. White arrowhead: yolk pools, black arrowhead: unfertilised oocytes, and open arrowhead: egg.

*C. elegans* hermaphrodites are protandrous, producing first sperm and then oocytes, and reproduction ceases by around day 4 of adulthood due to self-sperm depletion. While working with adult hermaphrodites expressing the vitellogenin (yolk protein) VIT-2 tagged with GFP (ref. ^10^), we noticed that older mothers leave patches of GFP-positive material on culture plates (Fig. 1a). Viewed under light microscopy these appeared as smears of a brownish substance (Fig. 1a). Venting of vitellogenin was confirmed and found to be highest on days 4–6 of adulthood, immediately after cessation of egg laying, and then to continue at lower levels until at least day 14 (Fig. 1b; Supplementary Fig. 1a). Yolk was vented through the vulva in brief bursts, either alone or with unfertilised oocytes (Fig. 1c, Supplementary Movie 1). Vented yolk also contains lipid, as shown by staining with a lipid dye (Fig. 1d; Supplementary Fig. 1b). Thus, cessation of egg laying due to self-sperm depletion is followed immediately by a burst of yolk venting.

### Vented yolk supports larval growth

**Fig. 2:**
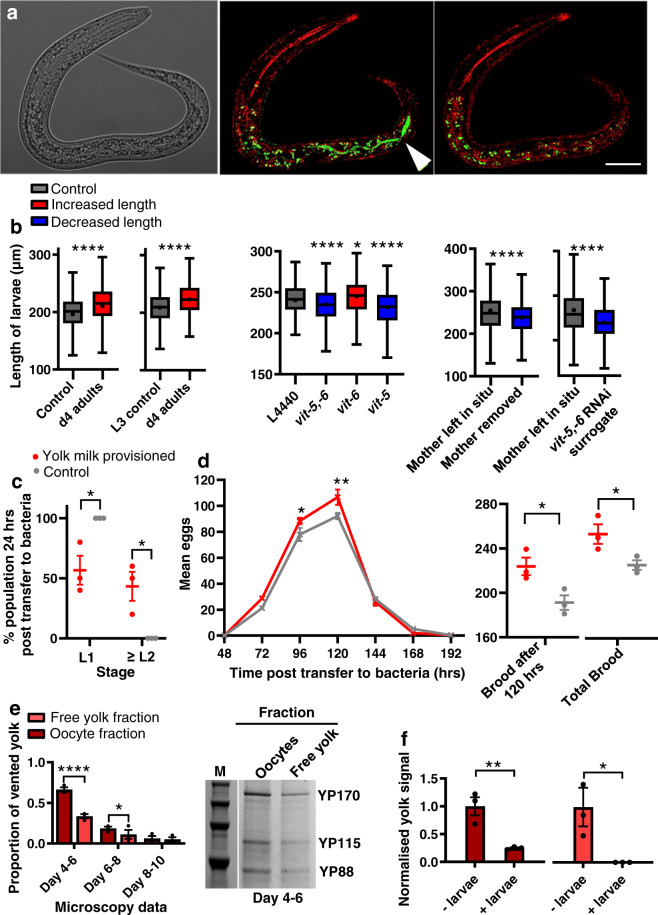
Vented yolk and unfertilised oocytes support larval growth. **a** Yolk in the intestinal lumen of a wild-type L1 larva after being left on a plate with no food apart from vented yolk (VIT-2::GFP labelled) for 4 h. Left: Nomarski microscopy image. Middle: larva imaged immediately after removal from plates using reflectance confocal microscopy (RCM) to highlight the refractive material of the terminal web that surrounds the intestinal lumen (fluorescence filters MBS T80/R20 and 405 nm excitation) (red) and superimposed airyscan image (488 nm excitation) (green) for GFP-labelled yolk. Right: same larva imaged 100 min later showing yolk no longer in the lumen, either due to defaecation or digestion; this disappearance confirms that the green fluorescence indicated in the central panel corresponds to yolk within the intestinal lumen. White arrowhead: VIT-2::GFP. Scale 20 μm. Fluorescence outside intestinal lumen is from gut granule autofluorescence^51^. For details on RCM, see Supplementary Fig. 2b, c. **b** Tukey box plots of length measurement of L1 larvae (line at median; + at mean; box limits are 25th and 75th percentiles; whiskers denote 1.5 times the interquartile range). Left and middle: larvae left from the egg stage for 48 h on plates preconditioned with day 4 adults left to vent for 24 h. L4440 empty vector control for RNAi-treated adults. Right: day 3 adults left to lay last few eggs and either left with larvae to vent yolk for 48 h or removed, or replaced with RNAi-treated surrogate mothers. Combined data of 3 trials (*n* = 200 worms per trial). Non-parametric two-tailed Kolmogorov–Smirnov tests were performed and corrected according to FDR. Left to right for stars *P* = <0.0001, <0.0001, <0.0001, 0.019, <0.0001, <0.0001, <0.0001. Red: treatments that increase the dependant variable; grey: control; and blue: that reduce the dependant variable. All larvae in trials are wild type. Larval cross-sectional area as well as length is increased following exposure of larvae to plates preconditioned with vented yolk (Supplementary Fig. 2d). **c** Recovery of larvae to the L2 stage and beyond, 24 h post transfer to bacteria. Two-way ANOVA (Bonferroni’s multiple comparisons test). Left to right for stars *P* = 0.014, 0.014. **d** Lifetime reproductive schedule. Left: Two-way ANOVA (Bonferroni’s multiple comparisons test); left to right for stars *P* = 0.049, 0.003. Right: unpaired two-tailed *t*-test; left to right for stars *P* = 0.049, 0.034. **c**, **d** Mean ± S.E.M. of 3 trials displayed (*n* = 10 worms per trial). Red: larvae transferred to bacteria after they were left from the egg stage for 48 h on plates preconditioned with day 4 adults left to vent for 24 h. Grey: control with larvae transferred to bacteria after they were left from the egg stage for 48 h on untreated plates. For data showing larval development on bacteria 48 h post transfer and time of first egg lay see Supplementary Fig. 2e. **e** Relative levels of free yolk vs yolk in unfertilised oocytes quantitated from VIT-2::GFP fluorescence on plates (*n* = 10 worms per trial). Data normalised to total yolk on days 4–6. Mean ± S.E.M. of 3 trials displayed. Two-way ANOVA (Bonferroni’s multiple comparisons test). Left to right for stars *P* = <0.0001, 0.037. Right: Protein gel showing YP bands after yolk collection from plates on d4–6. M: marker. For protein gel data of vented free yolk on all days see Supplementary Fig. 2f. **f** Larvae consume vitellogenin in both free yolk and oocyte fractions. Quantitated levels of YP170 from plates preconditioned with 100 venting d4 adults for 24 h followed by addition of 200 larvae or no larvae controls for 48 h. Oocytes and free yolk were washed from plates with M9 containing 0.001% NP-40 to solubilise vitellogenin^34^. Yolk and oocytes were then separated by gentle centrifugation, with the oocytes clearly visible in the pellet fraction. The two fractions were then subjected to protein gel electrophoresis. Data shows S.E.M. of 3 trials normalised to no larvae controls. Unpaired two-tailed *t*-test. Left to right for stars *P* = 0.009, 0.046. For protein gel data, see Supplementary Fig. 2g. *P < 0.05, ****P < 0.0001.

Given that yolk is a nutrient substance, one possibility is that vented yolk supports larval growth. Consistent with this, GFP-labelled yolk was observed in the intestinal lumen of larvae, indicating that yolk can be ingested (Fig. 2a). Notably, pre-treatment of *E. coli*-free agar plates with post-reproductive, 4-day-old mothers enhanced growth of L1 larvae, relative to control plates (no pre-treatment, or pre-treatment with non-venting L3 larvae; Fig. 2b). Moreover, subjecting mothers to *vit-5,-6* RNAi, which prevents vitellogenin accumulation^3^ also blocked the benefit to wild-type larval growth of plate pre-conditioning with venting mothers (Fig. 2b). *vit-5* RNAi decreases levels of the YP170 vitellogenin species but RNAi of *vit-6* (which encodes YP115/YP88) increases them^5^. These treatments, respectively, suppressed and enhanced effects of pre-treatment with venting mothers on larval growth (Fig. 2b), providing further evidence that larval growth is enhanced by consumption of vented yolk.

Next we tested whether yolk vented by mothers can benefit their own larvae. Fully-fed 3-day-old mothers were washed, placed on *E. coli*-free plates and left to lay their last eggs, and then either left in situ to vent yolk, or removed. Removal of mothers reduced growth of their progeny (Fig. 2b). In a further test, mothers were replaced after egg laying with other, surrogate mothers of the same age but treated with *vit-5,-6* RNAi, and this abrogated the benefit to larval growth (Fig. 2b). Taken together, these findings show that after self-sperm depletion *C. elegans* mothers can enhance growth of their offspring by venting yolk through the vulva, at least when other food sources are unavailable.

The enhancement of larval growth by yolk feeding implies an increase in larval fitness. Supporting this conclusion, yolk feeding also accelerated the resumption of normal growth in larvae transferred to an *E. coli* food source (Fig. 2c), and led to earlier reproduction and a larger brood size (Fig. 2d). As a means of transferring resources from mother to offspring after egg laying, vented yolk serves a function similar to that of mammalian milk; we therefore propose the term *yolk milk* to describe vented yolk, and also the accumulated yolk in the body cavity and uterus prior to venting. That yolk milk is beneficial to larvae led us to wonder whether larvae might be attracted to it; however chemotaxis tests revealed no such attraction, although larvae were attracted to adult hermaphrodites (Supplementary Fig. 2a). These findings reveal a surprising additional function for the intestine, uterus and vulva in *C. elegans* hermaphrodites: that of a form of primitive lactation.

### Yolk in unfertilised oocytes supports larval growth

After sperm depletion hermaphrodites lay over 100 excess, unfertilised oocytes, the overall volume of which exceeds that of the hermaphrodite herself, which has been noted as oddly wasteful and futile^11^. The timing of unfertilised oocyte production is similar to that of yolk venting (Fig. 1b). Indeed, yolk and unfertilised oocytes are often vented together (Fig. 1a) suggesting a possible role for vented oocytes in yolk milk transport. Consistent with this, vented oocytes contain large amounts of yolk, as shown by VIT-2::GFP, and confirmed by gel electrophoresis (Fig. 2e). At the outset of yolk/oocyte venting, oocytes contain twice as much vitellogenin as free vented yolk, but the proportion of the latter increases with age until day 10, when the ratio is 1:1 (Fig. 2e). To establish the extent to which vitellogenin delivered in each manner supports larval growth, free yolk and oocyte fractions were separated from conditioned plates on which L1 larvae had or had not been present for 24 h, and vitellogenin content was assayed. The results show that larval feeding causes a reduction in yolk from both the oocyte and free yolk fractions (Fig. 2f), i.e. vitellogenin in both free yolk pools and unfertilised oocytes is consumed by larvae. These results provide a possible explanation for the enigma of unfertilised oocyte production: that it represents an adaptation, aiding delivery of yolk milk to hungry young larvae.

### Insulin/IGF-1 signalling promotes yolk venting

**Fig. 3:**
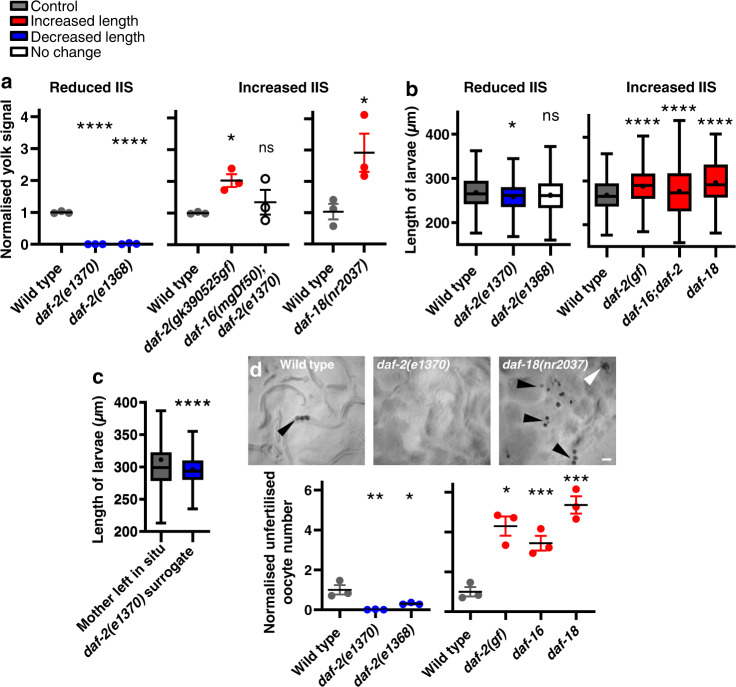
Yolk venting is promoted by insulin/IGF-1 signalling (IIS). **a** Yolk venting is downregulated by reduced IIS and upregulated by enhanced IIS. Quantitated YP170 band on protein gels. Means ± S.E.M. of 3 trials (*n* = 100 worms per trial). One-way ANOVA (Dunnett’s correction) or unpaired two-tailed *t*-test. Left to right for stars *P* = <0.0001, <0.0001, 0.049, 0.561 (ns), 0.046. **b** Decreased yolk milk provisioning by reduced IIS reduces larval growth on preconditioned plates relative to wild-type, and vice versa for increased IIS. Combined data of 3 trials (*n* = 200 worms per trial). Left to right for stars *P* = 0.019, 0.114 (ns), <0.0001, <0.0001, <0.0001. **c** Decreased larval growth on plates with *daf-2(e1370)* surrogate mothers. Wild-type mothers were allowed to lay their last eggs and either left in situ or replaced. *P* = <0.0001. **b**, **c** Tukey box plots of length measurement of L1 larvae (line at median; + at mean; box limits are 25th and 75th percentiles; whiskers denote 1.5 times the interquartile range). Non-parametric Kolmogorov–Smirnov two-tailed tests performed and corrected according to FDR. **d** Unfertilised oocyte production is downregulated by reduced IIS and upregulated by increased IIS. Bottom: Number laid for 24 h on d4–6 of adulthood and normalised to wild type. Means ± S.E.M. of 3 trials (*n* = 10 worms per trial). One-way ANOVA (Dunnett’s correction) or unpaired two-tailed *t*-test. Left to right for stars *P* = 0.005, 0.033, 0.002, <0.0001, <0.0001. Top: NGM plates imaged after 15 wild-type, *daf-18(nr2037)* or *daf-2(e1370)* worms were left for 24 h. Black arrowheads: unfertilised oocytes; white arrowhead: yolk pool. Red: treatments that increase the dependant variable; blue: that reduce the dependant variable; white: no effect; and grey: control. Scale 100 μm. *P < 0.05, **P < 0.01, ***P < 0.001, ****P < 0.0001.

*daf-2* insulin/IGF-1 receptor mutants are long lived^12,13^, and show reductions in vitellogenin synthesis^5,14^, PLP accumulation^3^ and unfertilised oocyte production^15^. Thus, IIS promotes ageing and production of yolk and oocytes. To test whether IIS promotes yolk milk venting we examined *daf-2* mutants, and found that yolk venting and promotion of wild-type larval growth by post-reproductive mothers was strongly reduced (Fig. 3a–c). Conversely, the *daf-2(gk390525)* gain-of-function (gf) mutation^16^ increased yolk venting and the resulting promotion of larval growth (Fig. 3a, b). Effects of *daf-2* on senescent pathology and lifespan require the *daf-16* FOXO transcription factor^3,12^. The *daf-16(mgDf50)* null mutation was found to restore to *daf-2* mutants both yolk venting and resultant promotion of larval growth (Fig. 3a, b). Moreover, mutation of the *daf-18* PTEN phosphatase^17^, which increases phosphatidylinositol (3,4,5)-trisphosphate (PIP_3_) signalling, increased yolk venting and larval growth (Fig. 3a, b). *daf-2(e1370)* surrogate mothers also failed to enhance growth of wild-type larvae hatched from final eggs (Fig. 3c; cf Fig. 2b right). In addition, suppression of unfertilised oocyte production by *daf-2(e1370)* is *daf-16* dependent^15^, and numbers of unfertilised oocytes laid were increased by both *daf-2(gf)* and *daf-18(nr2037)* (Fig. 3d). These results imply that IIS promotes *C. elegans* lactation, through promotion of *vit* gene expression and of gut-to-yolk biomass conversion^3^; whether IIS promotes yolk milk and oocyte venting solely by promoting their production, or whether it also exerts neuromuscular effects that promote their active release by the vulva remains unexplored. Thus, reduced IIS in *daf-2* mutants reduces later-life contributions to reproductive fitness and their associated costs, i.e. yolk milk production contributes to *C. elegans* senescence (particularly intestinal atrophy^3^).

### Yolk milk contains multiple IIS-regulated proteins that accumulate with age

**Fig. 4:**
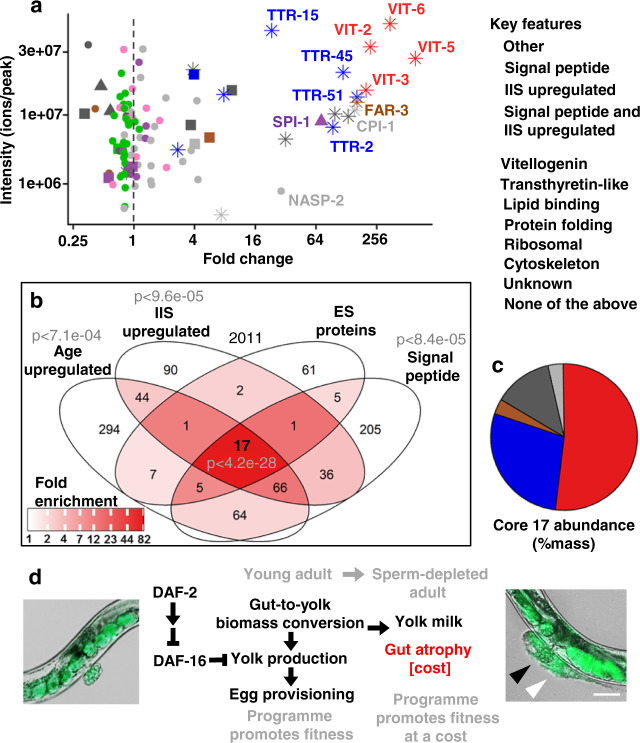
Analysis of adult ES proteome, including yolk milk proteome. **a** Relationship between protein level (intensity) in the day 4 adult ES proteome and increased abundance with age in the overall proteome, the latter from published data for wild type and *daf-2(e1370)* mutant adult *C. elegans* (ref. ^46^). “Age upregulated” was defined as showing an increase of >log2 = 1 day 17 vs day 1 (vertical dotted line). Right hand cluster includes many IIS- and age-upregulated secreted proteins. Left hand cluster likely includes more proteins shed by tissue breakdown. **b** Enrichment analysis of adult-specific ES proteome proteins relative to all proteins detected in the reference study^46^. Significant over-representation was detected using a one-tailed SuperExactTest^45^. **c** Percentage abundance categorisation of the core 17 proteins in the adult-specific ES proteome that are IIS upregulated, age upregulated and likely to be secreted (i.e. bear a predicted N-terminal signal peptide). For raw MS data see Supplementary Data 1. For details of IIS upregulation and *daf-16* dependence see Supplementary Fig. 3. For L3 ES proteome and specific proteins of interest see Supplementary Fig. 4. For tissue enrichment analysis and comparison to proteomic analysis of human milk see Supplementary Fig. 5. Data are available via ProteomeXchange with identifier PXD025472. **d** Model of intestinal conversion to yolk in sperm-depleted adults, where the benefit of yolk milk comes at the cost of intestinal atrophy. Here IIS promotes self-destructive somatic biomass repurposing, thus promoting senescence in a fashion typical of organisms exhibiting semelparous reproductive death^8^. Scale 50 μm.

**Fig. 5:**
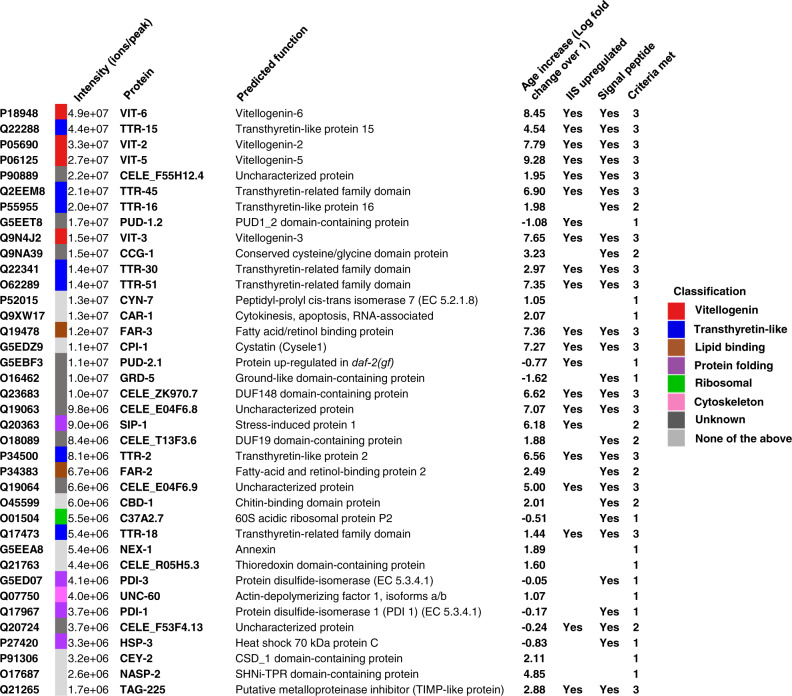
d4 adult-specific ES proteins. Those shown are either IIS upregulated, age upregulated or have a predicted N-terminal signal peptide. Significant differences in the distributions were detected using a Kolmogorov–Smirnov two-tailed test.

Our findings imply that the vitellogenin-rich fluid vented by sperm-depleted *C. elegans* functions as a milk. To further characterise *C. elegans* yolk milk we subjected it to proteomic analysis. Proteins released into media by adult hermaphrodites are expected to include not only vulvally-vented proteins but also proteins excreted via the excretory pore and anus, and shed from the nematode surface. Utilising terminology standard for the study of products released by parasitic helminths^18^, together these constitute the *C. elegans* adult excretory-secretory (ES) products. ES was collected from L3 larvae and hermaphrodites on day 4 of adulthood and analysed. The set of proteins present in the latter but not the former represents an adult-specific ES protein set that includes vulvally-vented proteins. A further expectation is that ES products will include both proteins of functional significance (e.g. yolk milk proteins), and random proteins shed from internal organs. Our analysis defined a set of 125 adult-specific ES proteins. Given that vitellogenins are secreted proteins whose levels increase with age in an IIS-dependent fashion, we focused on proteins that were (a) IIS regulated, (b) more abundant with increasing age and (c) putatively secreted (possessing a predicted N-terminal signal peptide). Overall, 21 ES proteins proved to be IIS upregulated, 30 upregulated in old age, and 28 to contain signal peptides (Figs. 4a, b and 5). Of these, 17 proteins exhibited all 3 features (Fig. 4c). This is 82-fold more than expected by chance alone and considering the frequency of each feature among all *C. elegans* proteins; and 6-fold more when also taking into account the overlap between the sets of proteins defined by each of the three features. We conclude that IIS promotes the production of certain proteins secreted from older worms whose abundance increases with age, defining a proposed IIS-activated, core yolk milk proteome.

Given that vitellogenins originate from the intestine, we wondered whether this was typical of adult ES proteins. To assess this we tested for over-representation of adult ES genes among tissue-specific gene expression sets. In fact, significant enrichment among intestinally expressed genes was not seen (*p* = 0.19), but this may be because around half of the genes in the *C. elegans* genome are intestinally expressed. However, over-representation was seen for genes expressed in several other tissues and cell types (Supplementary Fig. 5a). Notably, enrichment of adult ES proteins but not core yolk milk proteome proteins was detected in anal sphincter muscle- and anal depressor muscle-expressed genes (Supplementary Fig. 5a, b), potentially reflecting proteins shed via the anus.

Aside from the vitellogenins, which were the most abundant proteins, there were also several transthyretin-related (*ttr*) proteins, including TTR-2, -15, -16 and -51. TTRs often function as carrier proteins for lipophilic compounds^19,20^. Also present were FAR-3, another predicted lipid-binding protein, that is expressed in the vulva, as well as others like FAR-2 and LBP-6. Lipid carrier proteins are also abundant in mammalian milk, suggesting possible functional similarities. To probe further for possible similarities with mammalian milk, we compared the *C. elegans* adult ES with the human milk proteome^21^. Cross-species gene set analysis found a significant overlap between the two proteomes (*p* = 0.00146) (Supplementary Fig. 5c). Heat shock proteins (HSPs) sometimes contaminate protein extracts prepared for profiling, raising a concern that this statistically significant overlap might be artefactual; however, after exclusion of the two ES HSPs, the overlap remained statistically significant (*p* = 0.033). Moreover, at the protein category level, genes with Interpro terms associated with lipid binding were enriched in both cases (Supplementary Fig. 5d). These results suggest a modest level of similarity between *C. elegans* ES and the human milk proteome, despite the relatively distinct evolutionary origins of yolk and milk^22^.

## Discussion

In this study, we show that post-reproductive *C. elegans* hermaphrodites exhibit a form of primitive lactation, releasing yolk milk (free and in oocytes) through their vulva that can enhance growth and reproduction of progeny. We suggest that this provides a fitness benefit, coupled to pathological changes to reproduction-associated organs, in a process akin to reproductive death as seen in semelparous organisms (Fig. 4d). This coupling particularly affects the intestine, where self-destructive repurposing of biomass into yolk occurs^3^; notably, yolk synthesis in and secretion from muscle was also recently described^23^, raising the possibility that age-linked muscle atrophy in *C. elegans*^1^ also involves biomass repurposing. The occurrence of reproductive death in *C. elegans* is supported by comparative analysis with other *Caenorhabditis* species which shows that reproductive death is promoted by the germline in hermaphrodites (which vent yolk and oocytes) but absent in unmated females (which do not)^24^, and by comparison with other organisms that exhibit reproductive death^8^. We have also described elsewhere how reproductive death can facilitate the evolution of programmed adaptive death, which further shortens lifespan^25,26^.

Yolk milk feeding of larvae by mothers is a previously undescribed feature of *C. elegans* life history. Such maternal care by nematodes might seem surprising, but milk feeding occurs in other invertebrates, including tsetse flies (*Glossina* spp.)^27^ and the Pacific beetle cockroach *Diploptera punctata*^28^ (and in each case the nutrient fluid is referred to as milk). Yolk milk feeding by *C. elegans* also resembles trophallaxis, the transfer of food or nutritious fluids between individuals in a community, e.g. in social insects^29^. It is likely that in the wild, reproducing *C. elegans* exist largely as colony-like, high density, clonal populations that experience boom and bust population dynamics^25,30^. Potentially, it is into this collective that sperm-depleted hermaphrodites vent yolk milk. This would imply that natural selection favours lactation in *C. elegans* due to increases in inclusive fitness (through kin selection) rather than individual fitness.

Hermaphroditism facilitates rapid colonization of new food patches^30^, but protandry leaves mothers unable to contribute to fitness after sperm depletion. We suggest that later-life yolk milk production is an adaptation to circumvent this block to continued maternal contribution to fitness, here inclusive fitness of the surrounding clonal population. Plausibly, in *C. elegans* colonies fitness benefits of yolk milk venting will become significant only as microbial food supplies dwindle; this is in contrast to mammalian lactation, where milk is the main food source for neonates. Lactation also provides a solution to the long-standing enigma of copious unfertilised oocyte production in *C. elegans*^11^. A second possible fitness benefit from continued yolk production after self-sperm depletion is to build up yolk stocks in preparation for possible future reproduction after mating with males; however, this is insufficient to explain the bulk venting of yolk and oocytes, and the massive and self-destructive effort expended on yolk production, particularly given the extreme rarity of males in wild populations^30^.

Ageing is thought to evolve partly because genes exhibit antagonistic pleiotropy (AP), exerting both beneficial and deleterious effects on fitness; if the latter only occur later in life, such AP genes may be favoured by natural selection, contributing to the evolution of ageing^31^. Previously, post-reproductive yolk production and the pathologies to which it is coupled have been interpreted as futile run-on of reproductive function^1,3,5^. This is consistent with the AP theory, and recent ideas about its proximate mechanisms in programmatic terms, which argue that futile run-on of biological programmes in later life (or quasi-programmes) contributes to senescent pathology^31–33^. But if late-life yolk production contributes to fitness, then such production is not a quasi-programme but a programme proper, and pathologies such as gut atrophy are a direct cost of reproduction^34^. Thus, there is a trade-off between increased fitness due to yolk milk feeding of larvae (benefit) and intestinal senescence (cost) (Fig. 4d). These results provide a fresh account of the AP action of IIS pathway genes such as *daf-2* in which promotion of *costly programmes*^8^ provide inclusive fitness benefits.

## Methods

No statistical methods were used to predetermine sample size. The experiments were not randomised. The investigators were not blinded to allocation during experiments and outcome assessment. The work presented here complied with all relevant ethical regulations for animal testing and research.

### Culture methods and strains

*C. elegans* maintenance was performed using standard protocols^35^. Unless otherwise stated, all strains were grown at 20 °C on nematode growth media (NGM) with plates seeded with *E. coli* OP50 to provide a food source. An N2 hermaphrodite stock recently obtained from the Caenorhabditis Genetics Center was used as wild type (N2H) (ref. ^36^). Genotypes of most mutants used are as described in Wormbase (www.wormbase.org). Strains used included DR1296 *daf-2(e1368)*, GA114 *daf-16(mgDf50)*; *daf-2(e1370)*, GA1500 *bIs1 [pvit-2::vit-2::GFP* *+* *rol-6(su1006)]*, GA1928 *daf-2(e1370)*, GR1307 *daf-16(mgDf50)*, NS3227 *daf-18(nr2037)*, RT130 *pwIs23 [vit-2::GFP]* and PP2340 *daf-2(gk390525*).

### Live imaging and video capture of venting behaviour

In order to impede worm locomotion and facilitate imaging, polybead microspheres were used. d4 RT130 adults were placed on an NGM plate (no bacteria) in a 15–20 μl drop of 0.1 μm non-fluorescent polybead microspheres (2.5% solids [w/v] aqueous suspension with a coefficient of variance of 15%, 4.55 × 10^13^ particles/ml [Polysciences]). To remove any aggregated particles these were previously filtered through a 0.5 μm pore size syringe filter. A coverslip was then gently placed over the animal and observations made at ×200 magnification with Nomarski optics. To prevent fluorescence bleaching, Nomarski/GFP superimposed videoing and imaging were commenced only when movement of the vulval muscles was observed, which often preceded yolk milk venting or laying of eggs or unfertilised oocytes.

### Nomarski and epifluorescence microscopy imaging

Unless otherwise stated, live worms were placed onto 2% agar pads and anaesthetised using 0.2% levamisole. Images were captured using either a Zeiss Axioskop 2 plus microscope with a Hamamatsu ORCA-ER digital camera C4742-95 and Volocity 6.3 software (Macintosh version) for image acquisition; or an ApoTome.2 Zeiss microscope with a Hamamatsu digital camera C13440 ORCA-Flash4.0 V3 and Zen software. A constant exposure time was maintained between samples in fluorescence intensity comparisons. Brightness and contrast were adjusted equally across the entire image, and where applicable applied equally to controls. Where Nomarski and fluorescence images were superimposed, brightness and contrast were adjusted separately prior to superimposition.

### Confocal and airyscan imaging

For this, an inverted LSM880 microscope equipped with an Airyscan detector (Carl Zeiss, Jena) was used with a Plan-Apochromat 63 × 1.4 [numerical aperture (NA)] oil objective with a working distance of 0.19 mm. A 488 nm Argon laser was used for GFP excitation. In confocal mode, emission was recorded with an inbuilt GaAsP detector. For airyscan, the emission was recorded with the in-built 32-element GaAsP detector. For images showing sample change over time, images were only taken around every 50 min to prevent sample photobleaching. In the acquisition of 3D z-series, samples were imaged up to a sample depth of 41 μm, with images of 41 z-planes taken evenly through half of the diameter (dorsoventral) of the nematode. Data was processed using Fiji software (NIH), and the 3D Viewer plugin was used for 3D reconstruction. Brightness and contrast were adjusted equally across the entire image, and where applicable applied equally to controls. Where bright field and fluorescence images were superimposed, brightness and contrast were adjusted separately prior to superimposition.

### Reflectance confocal microscopy

This was performed using an inverted LSM880 microscope (Carl Zeiss, Jena) and a Plan-Apochromat 63 × 1.4 numerical aperture (NA) oil objective with a working distance of 0.19 mm. The main beam splitter was set to T80/R20 with multiphoton laser 405 nm excitation. Emission was recorded using an inbuilt GaAsP detector.

### Fluorescence quantitation of vented yolk milk

10 GA1500 L4 larvae were placed on 35 mm NGM plates (*n* = 5 plates per trial; total 50 worms) seeded with 100 μl *E. coli* OP50 and transferred every 24 h to new plates. After transferring, 5 superimposed GFP/Nomarski ×50 magnification images were taken of each plate at random positions across the bacterial lawn to sample approximately half of the lawn area. For each time point 3 control NGM plates (no worms) were treated in the same way and imaged.

Fluorescence quantitation of images was performed using the formulae below to account for varying levels of NGM background fluorescence in different plates and variations in fluorescence excitation. Yolk milk pools form localised regions of bright fluorescence on plates. Therefore the minimum emission fluorescence of an NGM plate can be used as an indicator of the true level of background fluorescence, since the range between the minimum and maximum background fluorescence of NGM is relatively constant between plates (data not shown). By measuring the background autofluorescence of control plates and normalising it to the minimum of treated plates, an accurate estimate of the range and maximum background fluorescence of treated control plates can be estimated and subtracted, using the following procedure (note: fluorescence calculations only refer to the GFP channel).

(i) Images with dust and/or cholesterol crystals in the agar (which can reduce the minimum emission fluorescence and affect the background fluorescence range) were manually censored (visualised under Nomarski as black dots or black crystals).
(ii) The ratio of minimum and maximum values of fluorescence intensity (FIR) is given by:
  1 $$\rho =\frac{{F}_{{{\max }}}}{{F}_{{{\min }}}}$$
  where ${F}_{{{\max }}}$ is the maximum emission fluorescence detected on a plate, and ${F}_{{{\min }}}$ the minimum emission fluorescence detected (both from background fluorescence; ${F}_{{{\min }}}$ to ${F}_{{{\max }}}$ = background fluorescence range).
(iii) The FIRs were averaged for the three control plates imaged each day to provide a daily control fluorescence intensity ratio $\bar{\rho }$ (DCIR). This also accounts for variation in UV lamp output during sample excitation. For each treated plate image, the DCIR was multiplied by the image ${F}_{{{\min }}}$ in order to set a threshold (T) fluorescence level on treated plates below which all fluorescence was considered background fluorescence and discounted.
  2 $${F}_{T}={F}_{{{\min }}}\bar{\rho }$$
(iv) Once done, the remaining fluorescence from above the threshold was isolated to ROIs containing GFP-labelled protein, with the fluorescence consisting of both GFP fluorescence as well as background fluorescence. Background fluorescence from these ROIs was removed as follows:
  3 $${F}_{{{{{{\rm{GFP}}}}}}}=-A{F}_{T}+\mathop{\sum} _{u}{F}_{u}\,A={{{{{\rm{Pixel}}}}}}\; {{{{{\rm{count}}}}}}$$
  where ${F}_{({{{{{\rm{GFP}}}}}})}$ is the total fluorescence from GFP on the plate, and ${F}_{u}$ is the fluorescence from a given ROI, with $\mathop{\sum}\limits_{u}{F}_{u}$ the total fluorescence from the collection of ROIs remaining on an image after step (iii).
(v) Separation of yolk and oocyte fluorescence was done manually for each image using Volocity 6.3 Acquisition. The free-drawing tool was used to identify oocytes on Nomarski/GFP images by viewing the Nomarski image, followed by subtracting all sum fluorescence within the ROIs from the total calculated in steps (i–iii).
  4 $${{F}_{{{{{{\rm{Freeyolk}}}}}}}=F}_{{{{{{\rm{GFP}}}}}}}-\mathop{\sum} _{u}{F}_{{{{{{\rm{Oocyte}}}}}}(u)}$$

### Gel electrophoresis and quantitation of vented yolk proteins

100–200 L4 larvae were maintained on 35 mm plates freshly seeded with 100 μl OP50. Following transfer each 24 h, yolk milk was washed off with 1 ml of M9 containing 0.001% NP-40 to solubilise vitellogenin^37^ and 2 μg/ml BSA as an external standard. This solution was pipetted onto plates and a polystyrene bacterial loop was then used to suspend all surface material, including the bacterial lawn, into the solution. The resulting suspension was pipetted out and into an Eppendorf tube. Inclusion of BSA served a dual purpose as a control for protein loss during sample collection from plates, and as a loading control for gel electrophoresis. Samples were spun at 2600 rpm for 10 min at 4 °C. The pellet was collected as the oocyte-containing fraction which, when viewed under a dissecting microscope, contained all oocytes, with no oocytes visible in the supernatant. The pellet also contained the majority of the *E. coli*. 500 μl of the supernatant was used for the free yolk milk analysis. Bacterial protein bands did not interfere with the detection of YP170 or BSA bands analysed (Supplementary Fig. 1a). Samples were then lyophilised with 10 μl glycerol, and the remaining pellet solubilised in a solution of 30 μl 4% SDS solution pH9 containing 12 mg bromophenol blue, 1 ml 1 M Tris HCL pH 8, and 10 μl 0.5 M EDTA (method optimised for YPs) by heating 2–4 times (based on the presence of a pellet post treatment) at 95 °C for 5 min while vortexing periodically, and finally centrifuged at 6000 rpm for 10 min. To prevent sample loss, all pipetting was performed using LoBind pipette tips and storage was in LoBind Eppendorf tubes. Sodium dodecyl sulphate–polyacrylamide gel electrophoresis (SDS-PAGE) was then performed, using Criterion XT Precast Gels 4–12% Bis-Tris (Invitrogen) and XT MOPS (Invitrogen) as a running buffer (7:1 ratio with Milli-Q water) at 90 V. Gels were stained with colloidal Coomassie blue as described^38^, using 5% aluminium sulphate-(14-18)-hydrate and 2% orthophosphoric acid (85%) to create colloidal particles. Gels were analysed using ImageQuant LAS 4000 (GE Healthcare). Protein band identification was based on published data^14^. Within lanes, vented YPs were normalised to the BSA that had been added during sample collection as an external standard.

### Staining of lipid in hermaphrodites prior to venting

For each time point, vital staining of worms was performed as described^39^ using the fluorescent dye BODIPY 493/503 (488 nm Exc./505–575 nm Em.) (Invitrogen) at a final concentration of 6.7 μg/ml, with hermaphrodites incubated in dye for 20 min at RT in darkness. Nematodes were then transferred to NGM plates (no *E. coli*) and allowed to crawl for 3–5 min to remove surface dye, followed by transfer to 35 mm NGM plates (no *E. coli*), with 20 worms per plate (1–2 plates per trial), and left to vent for 24 h. Fluorescence quantitation was performed as described for fluorescence quantitation of vented YPs, with the exception that Nomarski/fluorescence superimposed images were taken of the 5 regions with the most BODIPY fluorescence. This was due to the lower levels of BODIPY fluorescence relative to VIT-2::GFP fluorescence (likely due to differences in the fluorophores rather than the quantity of vented lipid vs protein). Control plates with L4 larvae (rather than adults) contained no patches of BODIPY fluorescence, ruling out a contribution of excess surface dye or egested dye to the observed BODIPY staining.

### Statistics and reproducibility

For all representative microscopy images and videos, each experiment was repeated independently with similar results at least three times.

### Determination of brood size and unfertilised oocyte number

L4 hermaphrodites were placed individually on 35 mm NGM plates with OP50 (*n* = 10 worms per trial), and worms transferred every 24 h until unfertilised oocyte production ceased. Eggs were left for 24 h to hatch and brood sizes counted. Clumps of unfertilised oocytes were carefully separated using a platinum pick to distinguish individual cells, using a dissecting microscope at maximum magnification.

### Chemotaxis assay

The chemotaxis assay was adapted from a previous study^40^. Briefly, a 5 cm NGM plate was divided into quadrants with test compounds or d5 adults placed on two opposite quadrants, and controls placed on the 2 remaining quadrants. Plates were then left for 4 h to form a chemotactic gradient. All spots also contained 4 μl of 0.25 M sodium azide as anaesthetic. Next, a 2 μl drop containing ~75 arrested L1 larva/μl was pipetted onto the centre of the plate within a 0.5 cm radius inner circle. Based on larval movement, a chemotaxis index (CI) was calculated using a formula as previously described^40^.

For experiments involving adult worms, a single, heat-killed d5 adult was used for the treatment with controls treated the same way but with no worm. For experiments involving vented yolk, sample collection was performed by concentration of 100 d4 adults (washed twice to remove surface bacteria) onto a small microwell cell-culture plate (Thermo Scientific Nunc 24-well cell-culture multidishes, 1.9 cm^2^) containing 500 μl NGM lacking bactopeptone (to make the agar harder and limit bacterial growth) and carbenicillin added topically 24 h prior to a concentration of 50 mM. This was done by picking worms in groups of 30–40 and holding them above the microwell, immediately after which a 10 μl drop of M9 was pipetted onto the pick to wash the worms into the well (no more than 30 μl of M9 was used per well). The worms were then allowed to vent for 24 h, after which a sterile scalpel was used to cut around the NGM well and transfer it to a 10 ml NGM plate right side up. Adults were left for 1 h to crawl off, after which remaining adults were picked off. The NGM with vented yolk was then cut into quadrants and each was placed as a treated section in a chemotaxis assay. Control sections were treated the same way but either had no worms, or L3 larvae added instead of d4 adults.

### Pre-conditioning of plates with vented yolk milk for larval growth assays

Thirty d4 adults were washed twice in M9 to remove surface bacteria and left to vent for 24 h on 35 mm NGM plates lacking bactopeptone, with 30 μl of 500 mM carbenicillin in Milli-Q water (plus 4 μl of 500 mM kanamycin in Milli-Q water for RNAi-treated adults as RNAi clones contain a carbenicillin/ampicillin resistance plasmid) added topically 24 h prior (2–3 plates per trial). Controls had no d4 adults or L3 larvae treated in the same way. Plates with bagging (i.e. with internally hatched larvae) or ruptured worms were censored. Next, 200 alkaline hypochlorite-treated eggs were placed on each plate and left for 48 h to develop, after which larval length was measured.

### Tests of yolk milk feeding by mothers of their own larvae

Shortly after reaching d3 of adulthood, 30 hermaphrodites were washed twice in M9 and placed on 35 mm bactopeptone-less NGM plates (2–3 plates per trial) and left for 24 h to lay their last eggs. Following this, the adults were either left in situ to vent yolk, removed, or replaced with surrogate mother worms of the same age. After 48 h all adults were removed and larval length measured. Plates with bagging or ruptured worms were censored.

### Larval length measurements

To collect larvae, plates were washed using 500 μl of M9 x 3 times and the collected liquid freeze-thawed (−80 °C) to kill and thereby straighten larvae. The samples were spun down at 2000 RPM at 4 °C for 5 min and the pellet along with 500 μl of liquid above it placed on slides for imaging. Volocity 6.3 software (Macintosh version) was then used to measure length.

### Proteomic analysis of d4 hermaphrodite excretory-secretory (ES) products

Three hundred and fifty fully-fed d4 hermaphrodites were picked onto NGM plates (no *E. coli*) and allowed to crawl for 30–60 s to remove surface bacteria before being transferred to a 500 μl solution of 0.001% NP-40 in M9 to prevent vented yolk from adhering to the nematode surface^37^. The worms were allowed to vent for 30 min (with picking of worms into each tube taking an additional 30 min) with the Eppendorf tubes maintained horizontally to aid diffusion of emitted proteins. The Eppendorf tube was placed upright for 1 min to allow nematodes to settle to the bottom of the tube, and 350 μl of solution drawn from the top of the tube. After collection, samples were carefully checked for the presence of adult worms and unfertilised oocytes, but none were observed. The adults worms were checked for the presence of ruptured animals, which were easy to identify, and where found the sample collected from that tube was discarded. ES products were also collected for L3 larvae, as a negative control for venting through the vulva (which is absent at this stage).

Independent samples were digested with trypsin and prepared for proteomic analysis^41^. Samples were analysed on a Thermo Scientific Q-Exactive Plus Orbitrap mass spectrometer connected to an Ultimate 3000 nanoLC system. Samples were trapped on a Thermo Scientific Acclaim PepMap C18 cartridge (0.3 mm × 5 mm, 5 μm/100 Å) and then chromatographed on a Thermo Scientific Easy-Spray Acclaim PepMap C18 column (75 μm × 15 cm, 3 μm/100 Å packing) eluting at 300 nl/min with a 30 min linear gradient of acetonitrile:water:formic acid (5:95:0.1–56:44:1 v/v/v). A full MS scan (*m*/*z* 135–2000 at 70,000 resolution) was acquired with a maximum injection time of 100 ms, and the 10 most intense ions with an intensity threshold 2.0e4 were selected for higher-energy C-trap dissociation (HCD) with a lock mass of *m*/*z* 445.12003. The normalised collision energy was 30, with an isolation width of 2 Da and dynamic exclusion of 20 s; singly charged ions were excluded. All chromatography solvents were Optima LCMS grade (Fisher Scientific). The mass spectrometry proteomics data have been deposited to the ProteomeXchange Consortium via the PRIDE^42^ partner repository with the dataset identifier PXD025472.

### Bioinformatic analysis of the d4 hermaphrodite ES proteome

Proteomes were quantified using MaxQuant 1.6.12.0 (ref. ^43^) with the default search settings and the *C. elegans* protein database from Uniprot (downloaded 20 December 2019), and downstream analysis was performed using the Proteus package in R (ref. ^44^). Proteins were considered detected in a sample if at least one proteotypic peptide from that protein was detected. Proteins were scored as present in the ES proteome if present in at least two of the three replicate samples. Proteins were only considered specific to the day 4 ES proteome if not detected in all three L3 control samples.

To estimate the composition of the ES proteome, proteins were manually classified according to name (e.g. vitellogenin, transthyretin) or known/predicted functions as indicated in WormBase (http://www.wormbase.org, release WS280) (see Supplementary Data 1 for classification of all proteins identified), and the sum of all peptide intensities across each grouping displayed. Significant over-representation of ES proteome proteins with respect to other datasets was detected using a one-tailed SuperExactTest^45^. To assesses whether the adult ES proteome showed any distinct patterns of expression in ageing worms or IIS knockdown (in *daf-2(e1370)* and *daf-16(mu86)* mutants, which are similar to *daf-16(mgDf50)* mutants used in other experiments), an existing proteome dataset^46^ was used. Proteins were considered upregulated in ageing worms if expression at least doubled from 1- to 17-day-old worms. Proteins were considered IIS-upregulated if expression in 17-day-old wild-type worms was at least twice the expression in 17-day-old *daf-2(e1370)* worms, normalising for any initial expression differences between 1-day-old wild-type and *daf-2(e1370)* worms. The presence of signal peptides in *C. elegans* proteins was predicted using SignalP 5.0 (ref. ^47^). In order to compare the composition of ES proteome proteins to human milk^21^, cross-species gene set analysis was performed using the XGSA package in R (ref. ^48^), accounting for protein homology mapping between *C. elegans* and humans. Tissue enrichment analysis was performed using the Wormbase tissue enrichment tool^49^ while GO term and Interpro term enrichment was performed using DAVID 6.8 (ref. ^50^).

### Reporting summary

Further information on research design is available in the Nature Research Reporting Summary linked to this article.

## Supplementary information

Supplementary information

Supplementary Data 1

Supplementary Data 2

Supplementary Data 3

Supplementary Data 4

Supplementary Data 5

Supplementary Data 6

Supplementary Movie 1

Reporting Summary

## Data Availability

The data that support the findings of this study are available from the corresponding author upon request. For mass spectrometry data shown, raw data are provided in Supplementary Data 1 and deposited to the ProteomeXchange Consortium via the PRIDE^42^ partner repository with the dataset identifier PXD025472.
