# Supplementary material for: C. elegans feed yolk to their young in a form of primitive lactation
Source: Nat Commun. 2021 Oct 5;12:5801. doi: 10.1038/s41467-021-25821-y (PMC8492707; doi:10.1038/s41467-021-25821-y)
Supplement: Supplementary file 1 — Supplementary information [file 41467_2021_25821_MOESM1_ESM.pdf]

Supplementary Information

C. elegans feed yolk to their young in a form of primitive lactation

Kern et al.

Supplementary Figure 1. Characterisation of yolk venting.

Supplementary Figure 2. Larvae are not attracted to vented yolk, show increased fitness upon yolk feeding, and consume both free yolk and yolk in oocytes.

Supplementary Figure 3. IIS-regulated proteins in the d4 adult-specific ES proteome.

Supplementary Figure 4. d4 adult-specific and L3 ES proteome comparison.

Supplementary Figure 5. d4 adult-specific ES proteome tissue enrichment analysis and comparison to the human milk proteome.

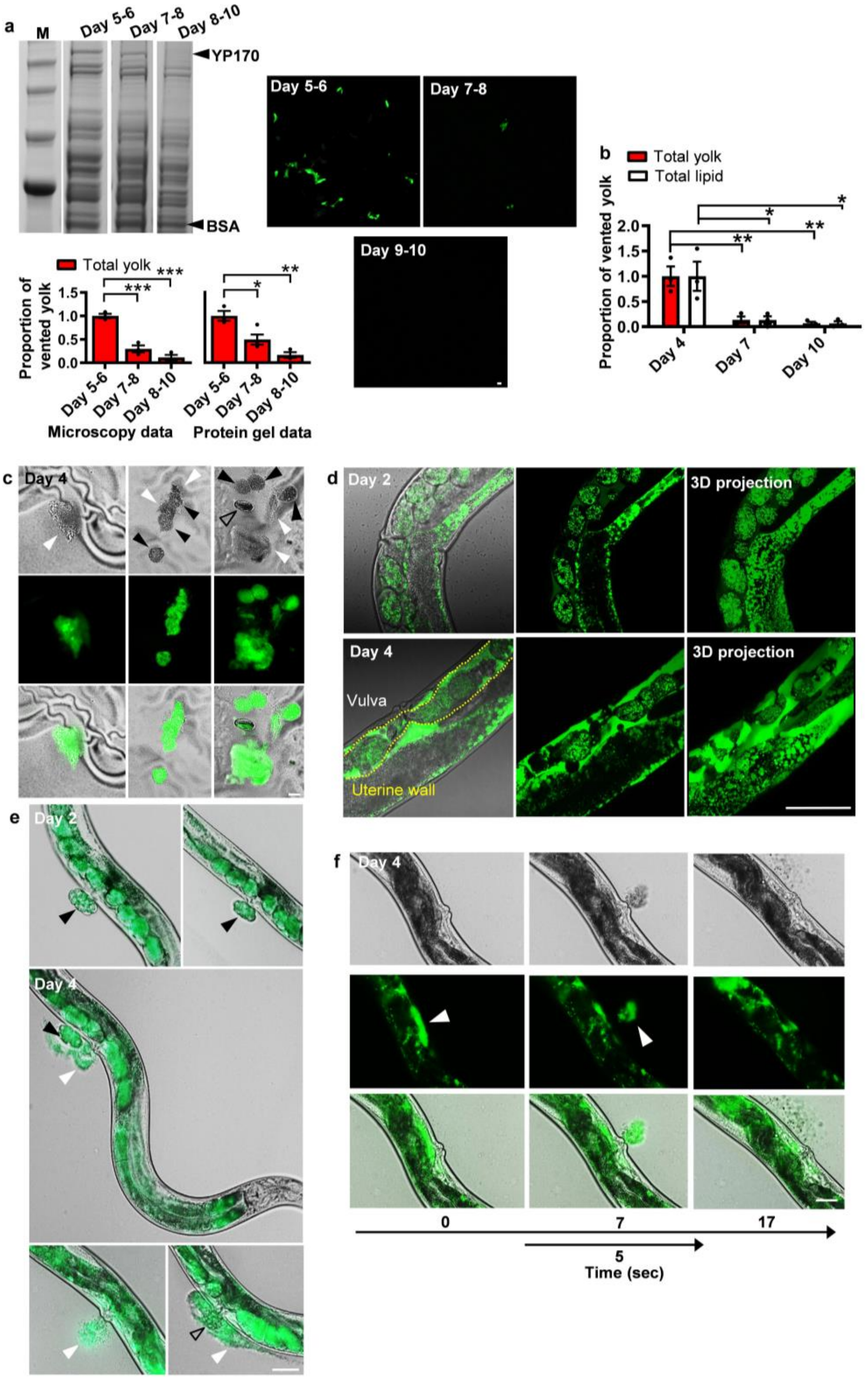

Supplementary Figure 1. Characterisation of yolk venting. **a**, Vented yolk on different days of adulthood. Left: Coomassie stained gel showing vented proteins (n=100 worms per trial); YP170 band is visible. BSA, external standard. M: marker. Other YP bands are too faint to be seen. Right: yolk patches left on plates by VIT-2::GFP d4 adults left for 24 hr (n=10 worms per trial). Scale 50 μm. Bottom: Relative levels of total yolk quantitated from VIT-2::GFP fluorescence on plate, or from protein gel analysis of YP170. Data normalised to total yolk on days 4-6. Mean ± s.e.m. of 3 trials displayed. One-way ANOVA (Tukey correction). Left to right for stars P= 0.000,

0.000, 0.018, 0.002. **b**, Lipid venting by BODIPY 493/503 vital stained adults follows the same pattern through time as vitellogenin venting by VIT-2::GFP adults. Values show mean ± s.e.m. of 3 trials (n=10 worms per trial). One-way ANOVA (Bonferroni correction). Left to right for stars *P*= 0.005, 0.029, 0.004, 0.021. **c**, Nomarski, GFP and superimposed images of vented yolk pools and unfertilised oocytes on NGM plates. Scale 50 µm. **d**, Yolk in the uterus of d4 adults but little/negligible yolk in the uterus of d2 adults. VIT-2::GFP animals. Left: internal yolk airyscan confocal and Nomarski superimposed images; middle: airyscan confocal image through mid-body of worm; and right: 3D projection of images of 41 Z-planes taken up to a sample depth of 41 µm through half the width of each worm. Scale 50 µm. **e**, Yolk venting on d4 compared with negligible yolk surrounding eggs laid on d2 of adulthood. Yolk venting on d4 may also be accompanied with the worm laying its last few fertilised eggs, or laying of unfertilised oocytes. Scale 50 µm. **f**, Nomarski, GFP and superimposed images of yolk venting through time. One venting burst takes approximately 5 sec; c.f. Supplementary Movie 1. Left: yolk seen in the uterus at time 0 sec, middle: yolk venting at time 7 sec, and right: less yolk in the uterus post venting at time 17 sec. Scale 50 µm. White arrowhead: yolk pools, black arrowhead: unfertilised oocytes, and open arrowhead: eggs. *P* *<0.05, *P* **<0.01, *P* ***<0.001.

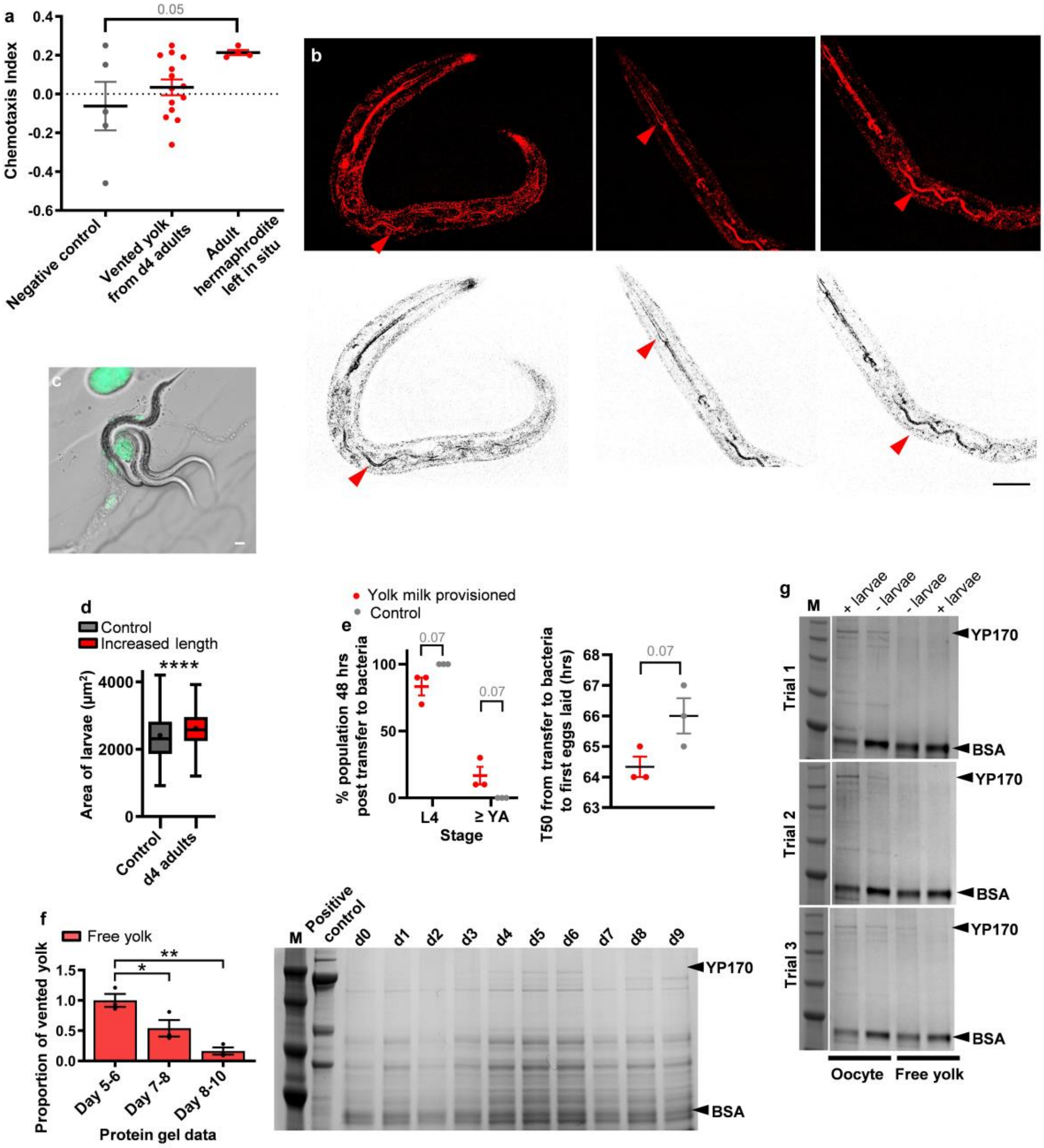

Supplementary Figure 2. Larvae are not attracted to vented yolk, show increased fitness upon yolk feeding, and consume both free yolk and yolk in oocytes **a**, Larvae are not attracted to vented yolk (but are attracted to adult hermaphrodites). Data show calculated chemotaxis index of individual trials (each dot represents one trial; n=75 larvae per trial) with mean ± s.e.m. displayed. One-way ANOVA. **b**, RCM used to highlight the dense terminal web that surrounds the intestinal lumen[1] (red arrow). Top: RCM image. Bottom: RCM image with colour inversion (dark values mapped to light and vice versa, without altering the grey distribution) to aid visualisation. Scale 20 µm. **c**, Larvae on plates with no food apart from vented yolk. Scale 50 µm **d,** Tukey box plot of cross-sectional area (accounting for larval width and length) of L1 larvae formed from eggs left for 48 hrs on plates preconditioned with day 4 adults left to vent for 24 hrs vs control plates (line at median; + at mean; box limits are 25th and 75th percentiles; whiskers denote 1.5 times the interquartile range). Combined data of 3 trials (n=200 worms per trial). Non-parametric Kolmogorov–Smirnov non-parametric two-tailed test; P= 0.000. Red: treatments that increase the dependant variable; grey: control. All larvae are wild type. **e**, Left: Time taken for 50% of the population (T50) to lay their first egg post transfer to bacteria. Unpaired two-tailed

t-test. Mean ± s.e.m. of 3 trials displayed (n=10 worms per trial). Right: Recovery of larvae to the L2 stage and beyond 48 hrs post transfer to bacteria. Two-way ANOVA (Bonferroni's multiple comparisons test). Mean ± s.e.m. of 3 trials displayed (n=10 worms per trial). Red: larvae transferred to bacteria after they were left from the egg stage for 48 hrs on plates preconditioned with day 4 adults left to vent for 24 hrs. Grey: Control with larvae transferred to bacteria after they were left from the egg stage for 48 hrs on untreated plates (c.f. Fig. 2c,d). **f**, Right: YP170 in free yolk protein vented by worms on different days of adulthood, and BSA standard (n=100 worms per trial). M: marker; Positive control: yolk protein from homogenised adult worms. Left: Relative levels quantitated. Data normalised to total yolk on days 4-6. Mean ± s.e.m of 3 trials displayed. One-way ANOVA (Tukey correction). Left to right for stars *P*= 0.033, 0.002. **g,** Larvae consume vitellogenin in both free yolk and oocyte fractions. Protein gel image of YP170 bands from 3 trials (c.f. Figure 2f). *P* *<0.05, *P* **<0.01, *P* ****<0.0001.

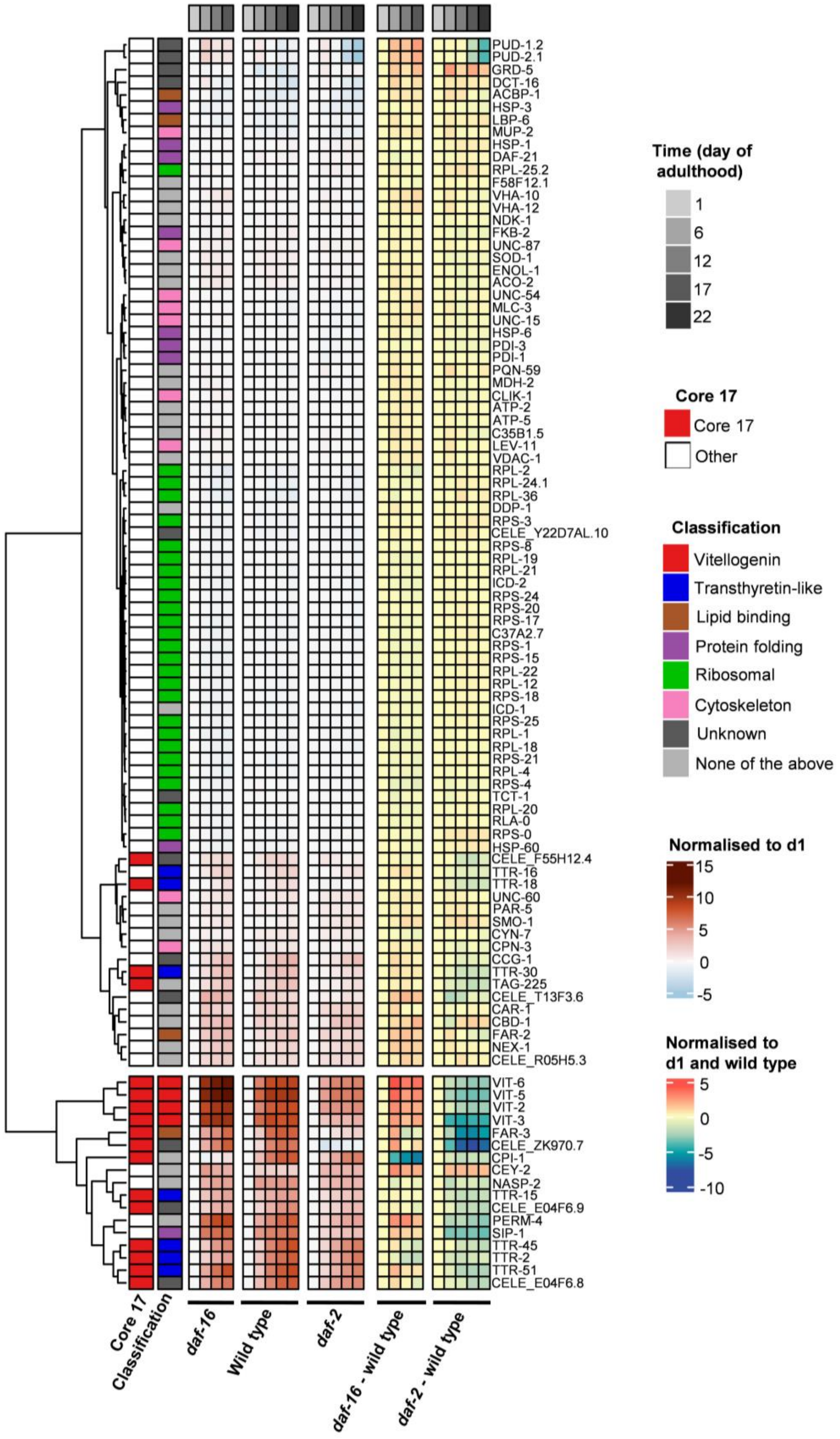

Supplementary Figure 3. IIS-regulated proteins in the d4 adult-specific ES proteome. Adult-specific ES proteome compared to published data[2] for age related changes in wild type, *daf-2(e1370)* and *daf-16(mu86)* mutants. Pairwise Manhattan distances of log2 SILAC ratios for wild type, *daf-2* and *daf-16* proteomes between days 1 and 22 were used to create a dissimilarity matrix, which was clustered using complete-linkage. Units of normalisation to day 1: log2 SILAC ratio - log2 SILAC ratio at day1. Units of normalisation to day 1 and wild type: (log2

SILAC ratio for mutant - log2 SILAC ratio for mutant at day 1) - (log2 SILAC ratio for wild type - log2 SILAC ratio for wild type at day 1).

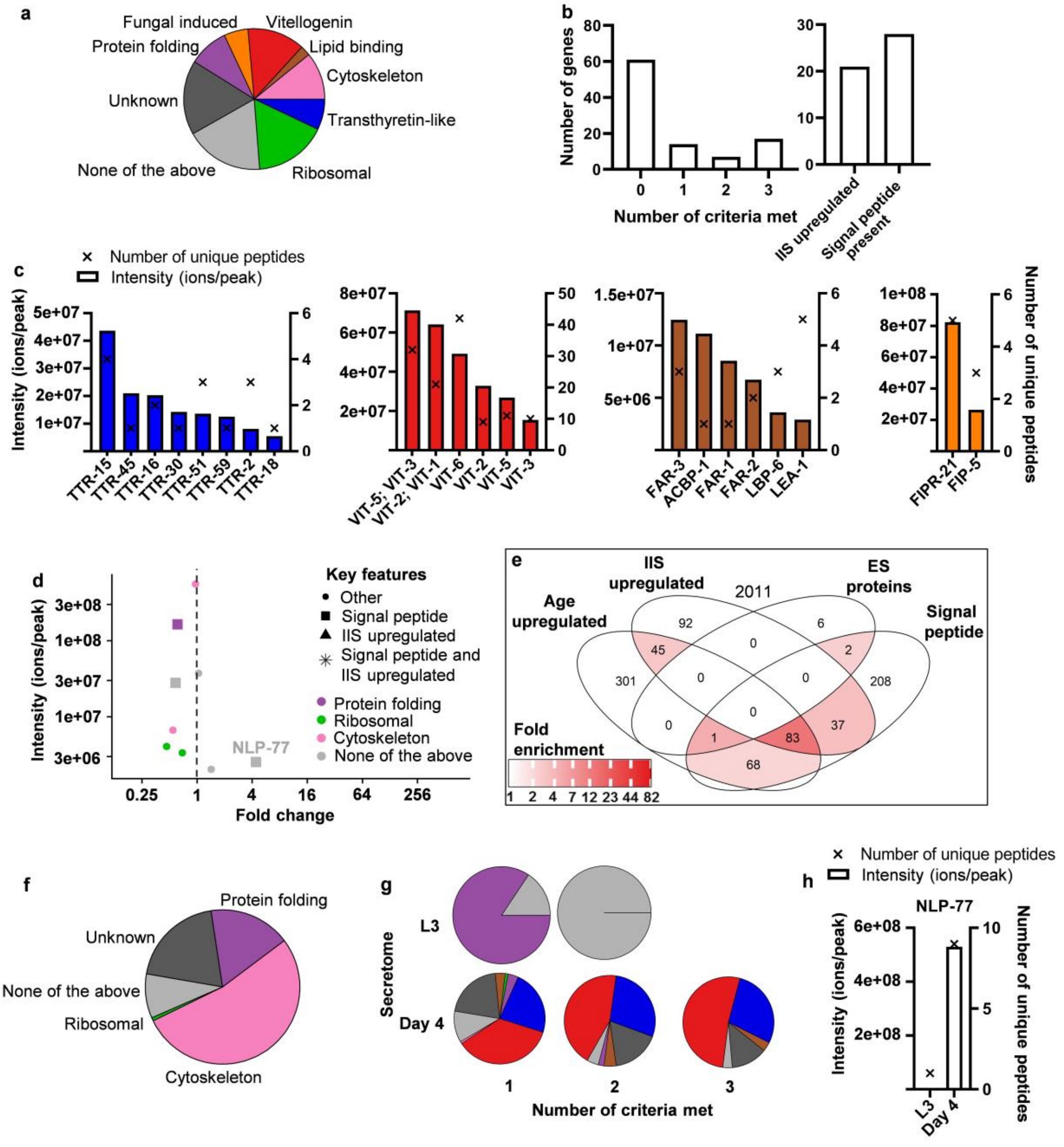

Supplementary Figure 4. d4 adult specific and L3 ES proteome comparison. **a**, All proteins detected in the adult-specific d4 ES proteome. Manual categorisation. **b**, Number of proteins in adult specific ES proteome meeting only 1, only 2 or all 3 criteria of being age upregulated, IIS upregulated or possessing an N-terminal signal peptide (c.f. Fig. 5), and total proteins that meet each requirement. **c**, Proteins of interest in the adult specific d4 ES proteome. Intensity and unique peptides of all vitellogenins, transthyretins, fatty acid binding proteins and fungal induced proteins present shown. Fungal-induced proteins were not detected in the internal proteome in a previous study[2]. The absence of VIT-4 unique peptides is likely an artefact resultant from the high sequence similarity between the vitellogenins: VIT-3 and -4 have more than 99% sequence similarity to each other[3]. A few proteotypic peptides were detected for VIT-4. **d**, Few proteins present in the L3 ES proteome. Intensity against internal proteome age associated upregulation displayed by comparison to published data[2]. **e**, No significant enrichment of proteins that are IIS upregulated and limited enrichment of age-upregulated proteins in the L3 ES proteome.

Enrichment analysis is relative to all proteins. Significant differences in the distributions were detected using a one-tailed SuperExactTest[4]. **f**, The L3 ES proteome primarily consists of cytoskeletal and protein folding associated proteins. Percentage abundance displayed. Manual classification. **g**, Percentage abundance categorisation of proteins in L3 ES proteome and adult specific ES proteome that meet either only 1, only 2 or all 3 criteria of being age upregulated, IIS upregulated or possessing a predicted N-terminal signal peptide. No proteins in the L3 ES proteome meet all 3 criteria. **h**, A highly abundant neuropeptide, NLP-77, with unknown function found in the d4 ES proteome and also found in smaller quantities in the L3 ES proteome, and so not classified as part of the adult specific d4 ES proteome.

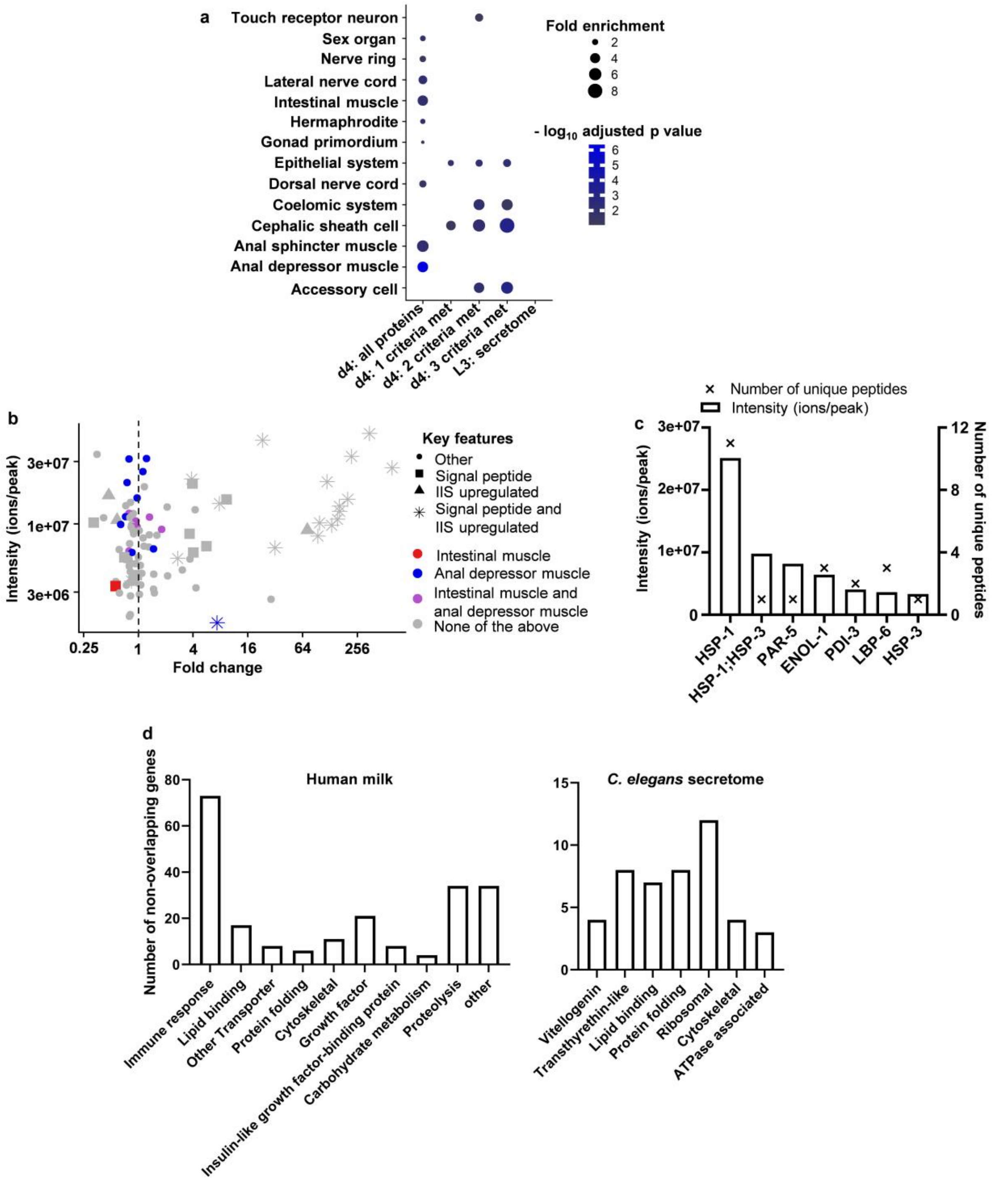

Supplementary Figure 5. d4 adult specific ES proteome tissue enrichment analysis and comparison to the human milk proteome. **a**, Tissue enrichment analysis of L3 and adult specific day 4 ES proteome. All proteins as well as those that met only 1, only 2 or all 3 criteria of being age upregulated, IIS upregulated or possessing a predicted N-terminal signal peptide are displayed. No enrichment was detected for the L3 ES proteome. Analysis was performed using the tissue enrichment analysis tool from WormBase[5]. One-tailed hypergeometric test. For exact P values see Supplementary Data 2. **b**, Most anal depressor muscle-related proteins are not IIS upregulated, age upregulated or N-terminal signal peptide-containing. **c**, Conserved proteins in human breast milk and the *C. elegans* day 4 adult specific ES proteome. Cross-species gene set

analysis performed using the XGSA package in R (Ref. [6]) accounting for homology mapping between *C. elegans* and humans. Human milk proteome from previously published data[7]. Overlap *P*=0.00146. Human homologues include: HSPA8 (heat shock protein family A (Hsp70) member 8); YWHAB (tyrosine 3-monooxygenase/tryptophan 5-monooxygenase activation protein beta); ENO1 (enolase 1); ENO2 (enolase 2); and ENO3 (enolase 3); PDIA3 (protein disulphide isomerase family A member 3); FABP7 (fatty acid binding protein 7); and HSPA5 (heat shock protein family A (Hsp70) member 5). **d**, Interpro term enrichment analysis relative to the full worm genome for the day 4 adult-specific ES proteome and comparison to human milk enrichment relative to human genome. Manual categorisation of groups with significant enrichment (cut-off of FDR *P*<0.05) is displayed to account for redundancy. Number of non-overlapping genes in each category is plotted. For full list of Interpro terms for *C. elegans* and humans see Supplementary Data 3 and 4, respectively. For full list of Gene Ontology Biological Process terms see Supplementary Data 5 and 6, respectively. Enrichment analysis was performed using Database for Annotation, Visualization and Integrated Discovery (DAVID)[8].

Supplementary references

1 McGhee, J. D. in *WormBook* (Ed The *C. elegans* Research Community) (2007).

2 Walther, D. *et al.* Widespread proteome remodeling and aggregation in aging *C. elegans*. *Cell* **161**, 919-932 (2015).

3 Spieth, J. & Blumenthal, T. The *Caenorhabditis elegans* vitellogenin gene family includes a gene encoding a distantly related protein. *Mol Cell Biol* **5**, 2495-2501 (1985).

4 Wang, M., Zhao, Y. & Zhang, B. Efficient test and visualization of multi-set intersections. *Sci Rep* **5**, 16923 (2015).

5 Angeles-Albores, D., Lee, R., Chan, J. & Sternberg, P. Tissue enrichment analysis for *C. elegans* genomics. *BMC Bioinformatics* **17**, 366 (2016).

6 Djordjevic, D., Kusumi, K. & Ho, J. XGSA: A statistical method for cross-species gene set analysis. *Bioinformatics* **32**, i620-i628 (2016).

7 D'Alessandro, A., Scaloni, A. & Zolla, L. Human milk proteins: an interactomics and updated functional overview. *J Proteome Res* **9**, 3339-3373 (2010).

8 Huang, D., Sherman, B. & Lempicki, R. Systematic and integrative analysis of large gene lists using DAVID bioinformatics resources. *Nat Protoc* **4**, 44-57 (2009).
