# Supplementary material for: C. elegans feed yolk to their young in a form of primitive lactation
Source: Nat Commun. 2021 Oct 5;12:5801. doi: 10.1038/s41467-021-25821-y (PMC8492707; doi:10.1038/s41467-021-25821-y)
Supplement: Supplementary file 9 — Reporting Summary [file 41467_2021_25821_MOESM9_ESM.pdf]

nature research

Corresponding author(s): David Gems

Last updated by author(s): Aug 24, 2021

Reporting Summary

Nature Research wishes to improve the reproducibility of the work that we publish. This form provides structure for consistency and transparency in reporting. For further information on Nature Research policies, see our Editorial Policies and the Editorial Policy Checklist.

Statistics

For all statistical analyses, confirm that the following items are present in the figure legend, table legend, main text, or Methods section.

n/a	Confirmed	
	x	The exact sample size (*n*) for each experimental group/condition, given as a discrete number and unit of measurement
	x	A statement on whether measurements were taken from distinct samples or whether the same sample was measured repeatedly
	x	The statistical test(s) used AND whether they are one- or two-sided<br>*Only common tests should be described solely by name; describe more complex techniques in the Methods section.*
	x	A description of all covariates tested
	x	A description of any assumptions or corrections, such as tests of normality and adjustment for multiple comparisons
	x	A full description of the statistical parameters including central tendency (e.g. means) or other basic estimates (e.g. regression coefficient) AND variation (e.g. standard deviation) or associated estimates of uncertainty (e.g. confidence intervals)
	x	For null hypothesis testing, the test statistic (e.g. *F*, *t*, *r*) with confidence intervals, effect sizes, degrees of freedom and *P* value noted<br>*Give P values as exact values whenever suitable.*
x		For Bayesian analysis, information on the choice of priors and Markov chain Monte Carlo settings
x		For hierarchical and complex designs, identification of the appropriate level for tests and full reporting of outcomes
x		Estimates of effect sizes (e.g. Cohen's *d*, Pearson's *r*), indicating how they were calculated

Our web collection on statistics for biologists contains articles on many of the points above.

Software and code

Policy information about availability of computer code

Data collection	Wormbase (www.wormbase.org, release WS280); Volocity 6.3 software (Macintosh version); Zen software (2);mageQuant LAS 4000 (GE Healthcare)
Data analysis	Wormbase (www.wormbase.org, release WS280); Fiji software (NIH); ImageQuant LAS 4000 (GE Healthcare); Volocity 6.3 software (Macintosh version); MaxQuant 1.6.12.0; Uniprot (downloaded 20 December 2019); Proteus package in R; SignalP (5.0); XGSA package in R; DAVID (6.8).

For manuscripts utilizing custom algorithms or software that are central to the research but not yet described in published literature, software must be made available to editors and reviewers. We strongly encourage code deposition in a community repository (e.g. GitHub). See the Nature Research guidelines for submitting code & software for further information.

Data

Policy information about availability of data

All manuscripts must include a data availability statement. This statement should provide the following information, where applicable:
- Accession codes, unique identifiers, or web links for publicly available datasets
- A list of figures that have associated raw data
- A description of any restrictions on data availability

The data that support the findings of this study are available in Supplementary Data 1 and deposited to the ProteomeXchange Consortium via the PRIDE partner repository with the dataset identifier PXD025472.

Field-specific reporting

Please select the one below that is the best fit for your research. If you are not sure, read the appropriate sections before making your selection.

[x] Life sciences   [ ] Behavioural & social sciences   [ ] Ecological, evolutionary & environmental sciences

For a reference copy of the document with all sections, see nature.com/documents/nr-reporting-summary-flat.pdf

Life sciences study design

All studies must disclose on these points even when the disclosure is negative.

Sample size	No sample size calculation was performed as trails were based on typically standard C. elegans experimental procedures
Data exclusions	No data were excluded
Replication	All attempts at replication were successful. All experiments were repeated at least 3 times
Randomization	Experiments were not randomized (noted in manuscript methods)
Blinding	Experiments were not blinded (noted in manuscript methods)

Reporting for specific materials, systems and methods

We require information from authors about some types of materials, experimental systems and methods used in many studies. Here, indicate whether each material, system or method listed is relevant to your study. If you are not sure if a list item applies to your research, read the appropriate section before selecting a response.

Materials & experimental systems

n/a	Involved in the study	
[x]	[ ]	Antibodies
[x]	[ ]	Eukaryotic cell lines
[x]	[ ]	Palaeontology and archaeology
[ ]	[x]	Animals and other organisms
[x]	[ ]	Human research participants
[x]	[ ]	Clinical data
[x]	[ ]	Dual use research of concern

Methods

n/a	Involved in the study	
[x]	[ ]	ChIP-seq
[x]	[ ]	Flow cytometry
[x]	[ ]	MRI-based neuroimaging

Animals and other organisms

Policy information about studies involving animals; ARRIVE guidelines recommended for reporting animal research

Laboratory animals	Caenorhabditis elegans: an N2 hermaphrodite stock recently obtained from the Caenorhabditis Genetics Center was used as wild type (N2H); and mutants included DR1296 daf-2(e1368), GA114 daf-16(mgDf50); daf-2(e1370), GA1500 bIs1 [pvit-2::vit-2::GFP + rol-6(su1006)], GA1928 daf-2(e1370), GR1307 daf-16(mgDf50), NS3227 daf-18(nr2037), RT130 pwIs23 [vit-2::GFP], and PP2340 daf-2 (gk390525).
Wild animals	Study did not involve wild animals
Field-collected samples	Study did not involve samples collected from the field
Ethics oversight	No ethical guidance was required

Note that full information on the approval of the study protocol must also be provided in the manuscript.
